# Exercise enhancement by RGS14 disruption is mediated by brown adipose tissue

**DOI:** 10.1111/acel.13791

**Published:** 2023-03-10

**Authors:** Dorothy E. Vatner, Marko Oydanich, Jie Zhang, Sara C. Campbell, Stephen F. Vatner

**Affiliations:** ^1^ Department of Medicine Rutgers, New Jersey Medical School Newark New Jersey USA; ^2^ Department of Cell Biology and Molecular Medicine Rutgers, New Jersey Medical School Newark New Jersey USA; ^3^ Department of Kinesiology and Health Rutgers University New Brunswick New Brunswick New Jersey USA

**Keywords:** aging, brown adipose tissue, exercise, RGS14 KO

## Abstract

Enhanced exercise capacity is not only a feature of healthful aging, but also a therapy for aging patients and patients with cardiovascular disease. Disruption of the Regulator of G Protein Signaling 14 (RGS14) in mice extends healthful lifespan, mediated by increased brown adipose tissue (BAT). Accordingly, we determined whether RGS14 knockout (KO) mice exhibit enhanced exercise capacity and the role of BAT in mediating exercise capacity. Exercise was performed on a treadmill and exercise capacity was assessed by maximal running distance and work to exhaustion. Exercise capacity was measured in RGS14 KO mice and their wild types (WT), and also in WT mice with BAT transplantation from RGS14 KO mice or from other WT mice. RGS14 KO mice demonstrated 160 ± 9% increased maximal running distance and 154 ± 6% increased work to exhaustion, compared to WT mice. RGS14 KO BAT transplantation to WT mice, resulted in a reversal of phenotype, with the WT mice receiving the BAT transplant from RGS14 KO mice demonstrating 151 ± 5% increased maximal running distance and 158 ± 7% increased work to exhaustion, at three days after BAT transplantation, compared to RGS14 KO donors. BAT transplantation from WT to WT mice also resulted in increased exercise performance, but not at 3 days, but only at 8 weeks after transplantation. The BAT induced enhanced exercise capacity was mediated by (1) mitochondrial biogenesis and SIRT3; (2) antioxidant defense and the MEK/ERK pathway, and increased hindlimb perfusion. Thus, BAT mediates enhanced exercise capacity, a mechanism more powerful with RGS14 disruption.

## Graphical abstract

It is well known that increased white adipose tissue (obesity) leads to decreased exercise capacity. The current investigation focused on brown adipose tissue (BAT) in the Regulator of G Protein Signaling 14 (RGS14) knock out mouse, which increases exercise capacity more than BAT in WT mice. The mechanisms by which RGS14 KO BAT increases exercise capacity through the SIRT3–MEK–ERK pathway, which results in increased mitochondrial function, decreased oxidative stress and increased angiogenesis, resulting in increased skeletal muscle blood flow, all working to mediate the increased exercise capacity in the RGS14 KO mouse.

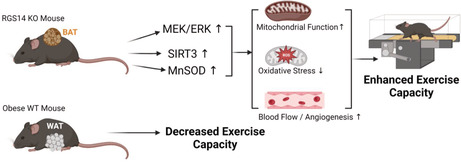

**Abbreviations**
3‐YTP: 3‐(1H‐1,2,3‐triazol‐4‐yl) pyridine
AC5: adenylyl cyclase type 5
ANOVA: one‐way analysis of variance
BAT: brown adipose tissue
EE: energy expenditure
GDI: guanine nucleotide dissociation inhibitor
GPCR: G protein‐coupled receptor
IRF4: interferon regulator factor‐4
KO: knockout
MAPK: mitogen‐activated protein kinase
MnSOD+/−: MnSOD heterozygous
Peak VCO2: peak carbon dioxide production
Peak VO2: peak oxygen consumption
RGS14: regulator of G protein signaling 14
SIRT3: sirtuin 3
WT: wild type

## 1 INTRODUCTION

While extending lifespan has been a major focus of modern medicine, the shift towards creating healthful, functional aging is becoming more vital. Exercise has been shown to protect against cardiovascular disease, cancer, depression, and obesity; conversely, reduction in exercise capacity is an early sign of disease (Brown et al., 2012; Craft & Perna, 2004; McQueen, 2009; Myers, 2003). The role of brown adipose tissue (BAT) mediating exercise performance and the mechanisms involved, are not known. It is known that exercise training promotes differentiation of white adipocytes to brown adipocytes, thereby enhancing overall BAT abundance in the trained individual (Agarwal, 2012; Cuevas‐Ramos et al., 2019). However, whether enhancing BAT amount and/or function is a novel mechanism to improve exercise capacity, the focus of this investigation, has not been studied previously. Disruption (genetic knockout or “KO”) of Regulator of G Protein Signaling 14 (RGS14) in mice is a model of healthful longevity, with increased BAT (Vatner et al., 2018), which improves metabolism and protects against cold exposure via a mechanism involving upregulation of the mitochondrial NAD‐dependent deacetylase sirtuin 3 (SIRT3).

The goals of the current investigation were, therefore, to examine the exercise capacity of RGS14 KO mice and whether increased BAT was responsible for the changes observed in exercise capacity. To accomplish these goals, we first examined the effects of BAT transplantation on exercise performance, by removing BAT from RGS14 KO mice and transplanting it into wild type (WT) littermate mice. To determine if RGS14 KO BAT was unique, we compared these data from RGS14 KO BAT transplants to exercise capacity in WT mice with BAT transplanted from other WT mice. We then examined mechanisms mediating the enhanced exercise capacity of RGS14 KO mice and WT mice with BAT transplants from RGS14 KO mice, including SIRT3, mitochondrial biogenesis, the MEK/ERK pathway, oxidative stress, exercise metabolism, and hindlimb blood flow. The role of BAT in mediating enhanced exercise performance was confirmed through transplantation experiments from RGS14 KO BAT to WT and from WT BAT to WT.

## 2 METHODS

### 2.1 Animal experimental procedures

All experiments were performed on 3–6‐month‐old RGS14 KO, RGS14 WT littermates, RGS14 KO × SIRT3 KO, SIRT3 KO, WT × MnSOD^+/−^, RGS14 KO × MnSOD^+/−^, BAT donor, and BAT recipient male mice. A subgroup of WT mice, with BAT transplanted from one WT to other WT mice, was also studied. The RGS14 KO mouse model was developed in our laboratory as previously described (Vatner et al., 2018). Briefly, RGS14 KO mice (RGS14tm1‐lex) were generated in the Transgenic Core Facility at Rutgers University – New Jersey Medical School, through the National Institutes of Health‐sponsored Mutant Mouse Regional Resource Center at http://www.informatics.jax.org/searches/accession_report.cgi?id=MGI:3528963. Embryos were implanted into C57/BL6 females, and founder mice were crossed with C57/BL6 to establish RGS14‐KO. All mice were from F1 heterozygote crosses. Genotypes were determined by PCR of genomic DNA from mice tails. WT forward primer was 5′ cagcgcatcgccttctatc 3′. Primer for the targeting vector was 5′ gcagcgcatcgccttctatc 3′ with a shared reverse primer 5′ agactggcagaagaattcagg 3′. SIRT3 KO mice were a gift from Dr. David Sinclair at Harvard Medical School (Hafner et al., 2010). The RGS14 KO × SIRT3 KO mouse model was generated by crossing RGS14^−/−^ and SIRT3^−/−^ (Vatner et al., 2018). The RGS14 KO × MnSOD^+/−^ mouse model was generated by crossing RGS14 KO mice with MnSOD^+/−^ mice. Mice were genotyped by standard PCR methods.

All mouse models were bred at Rutgers, New Jersey Medical School. For exercise studies, all mice were matched for body weight. Animals were all placed on standard chow and had free access to water for the duration of the study. All animals were kept on a standard 12:12 h light–dark cycle. These studies were approved by the Institutional Animal Care and Use Committee of Rutgers University—New Jersey Medical School. Animals were randomized and tested blindly for measurements listed below.

### 2.2 Brown adipose tissue removal and transplantation

Three–six‐month‐old male RGS14 KO mice or WT littermates were anesthetized using pentobarbital (60 mg/kg). The backs of the mice were shaved, and the mice were placed in the prone position. A 2‐cm midscapular transverse incision was made on the back of the mouse, and the BAT was freed from the surrounding muscles. Transplantation was initiated by removing BAT from RGS14 KO mice (RGS14 KO BAT donor). The removed BAT from donor mice was incubated in 10 mL saline at 37°C for 20–30 min. Three–six‐month‐old WT mice were anesthetized and 0.1 g of BAT from the RGS14 KO donor mice was subsequently transplanted into the visceral cavity of another WT mouse (RGS14 KO BAT recipient). The graft was carefully lodged deep between folds within the endogenous epididymal fat of the recipient mice. The skin incision in the recipient was closed with 6‐0 nylon sutures. The skin incision in the donor was closed using stainless steel wound clips. These mice were allowed to recover for 3 days prior to exercise testing. BAT transplantation was also done from donor C57BL6/J (WT) to recipient C57BL6/J (WT) mice as well as from donor RGS14 KO × SIRT3 KO double‐knockout mice to recipient WT mice. Whereas it took 3 days for WT mice with RGS14 KO BAT transplants to recapitulate the enhanced exercise performance of RGS14 KO donor mice, it required 8 weeks for WT mice with BAT transplants from other WT mice to reach enhanced exercise performance.

### 2.3 Exercise protocol and indices of exercise capacity

Mice were exercised on a treadmill (AN5817474; Accuscan Instruments) attached to a metabolic chamber to measure maximum exercise capacity. Mice were subjected to a practice trial 3 days before the experiment to adapt to the treadmill testing environment. Mice that underwent BAT transplantation were given a practice trial the day before exercise testing in order to allow for adequate recovery as well as treadmill environment acclimation.

Food was withdrawn 3 h before exercise testing. All mice were exercised at the same time of day for each experiment. All exercise testing was done by the same investigator, who was blinded to the genotype. At the time of the experiment, each mouse was placed on the treadmill with a constant 10% grade. The treadmill was started at 4 m/min, and the speed increased incrementally by 2 m/min every 2 min until the mice reached exhaustion. At the end of each treadmill lane was a rod that delivered a shock, which served as negative reinforcement for cessation of running. Exhaustion was defined as spending 10 seconds on the rod without attempting to reengage the treadmill belt. The indices of exercise capacity measured were maximal distance and work to exhaustion. RGS14 KO mice and WT littermate controls were separately treated with the SIRT3 inhibitor 3‐YTP (3‐(1H‐1,2,3‐triazol‐4‐yl) pyridine) (50 mg/kg i.p.) every 2 days for a total of 3 doses, as previously described (Shi et al., 2022; Ye et al., 2019), followed by exercise testing, as articulated above. For MEK/ERK inhibition, the MEK inhibitor U0126 (Sigma) was delivered to RGS14 KO mice and WT littermates at a dose of 10 mg/kg day^−1^ with a mini‐osmotic pump (ALZET model 2001; DURECT Corp) for 7 days, followed by exercise testing as previously described.

### 2.4 Indirect calorimetry

During exercise testing, mice were measured for peak oxygen consumption (peak VO_2_) and peak carbon dioxide production (peak VCO_2_); and energy expenditure (EE) were calculated by VCO_2_ and VO_2_ values. Measurements were taken once every minute during the exercise test and only the data point at maximal exhaustion was taken to represent peak VO_2_, VCO_2_, and EE.

### 2.5 Mitochondrial morphology

Mitochondrial number and cristae density were measured by electron microscopy. Tissues were perfusion fixed using a modified Karnovsky fixative (1.6% glutaraldehyde, 1.3% paraformaldehyde in 0.2 M sodium cacodylate buffer, pH 7.4). Skeletal muscles and BAT were cut into small cubes, post fixed in paraformaldehyde and osmium tetroxide, and embedded in Spurr resin. Mitochondria numbers and cristae density were determined using stereological procedures with the Philips/FEI CM12 transmission electron microscope (Texas, USA) under 8000× magnification for skeletal muscle and 12,000x magnification for BAT.

### 2.6 Hindlimb blood flow

Blood flow in the hindlimb was measured using non‐linear contrast imaging via the Vevo 3100 system (Visual Sonics Inc.). Animals were anesthetized using pentobarbital (60 mg/kg). Vevo MicroMarker contrast agents (Bracco Research SpA) were injected into each animal via an external jugular catheter. Each dose was 50 μL and contained 1 × 108 bubbles per dose. Each dose was delivered using an infusion pump (Harvard Instruments) and infused at a rate of 600 μL/min. Following infusion, blood flow, via the index of peak enhancement, a measure of peak blood flow, was calculated using VevoCQ software (Vatner et al., 2020).

### 2.7 Western blot analysis

Proteins separated by SDS–PAGE were transferred to nitrocellulose membranes. The membranes were probed with the primary antibody at 4°C overnight. The bands were visualized using chemiluminescence reagents. The linear range of detection for different proteins and band intensities were determined by densitometry. Blots were reprobed with GAPDH to control for sample loading. The antibodies used were VEGFA (46154; Abcam), SOD2/MnSOD (13534; Abcam), ERK1/2 (9102; Cell Signaling), pERK (9101; Cell Signaling).

### 2.8 Histology staining

BAT tissues were collected from RGS14 KO mice and WT mice fixed in 10% formalin. After embedding in paraffin, BAT samples were sectioned at 5 μm thickness and processed for Hematoxylin and Eosin staining (Petruzzelli et al., 2014; Sakaguchi et al., 2017). Skeletal muscles were freshly collected from WT, RGS14 KO, RGS14 KO BAT recipient, and RGS14 KO BAT donor mice, and embedded as OCT frozen blocks for capillary density and arteriole density staining. Hematoxylin and Eosin staining was used to visualize the overall BAT morphology and architecture for cell size and cell number. The images were captured at a field lens magnification of ×40 with 15 fields from each animal analyzed. Cell size and cell number were measured and counted in ImageJ using digital calipers at a field lens of ×40. Using isolectin staining (isolectin GS‐IB4‐Alexa448 conjugate, catalog # 1780254, Invitrogen), capillaries were identified as a single endothelial cell layer with a diameter less than 25 μm. Capillary density was quantified at 40× magnification as absolute number per unit (Vatner et al., 2020). Arteriole density was identified using smooth muscle actin (catalog # 9106S1102A; Dako). Arteriole density was quantified at 40× magnification as absolute number per unit (Vatner et al., 2020).

### 2.9 Statistical analyses

All data are expressed as mean ± SEM. To compare two independent groups, we used the Student's unpaired *t*‐test, whereas for more than two groups, one‐way analysis of variance (ANOVA) was used. *p* < 0.05 was taken as the level of significance.

## 3 RESULTS

**FIGURE 1:**
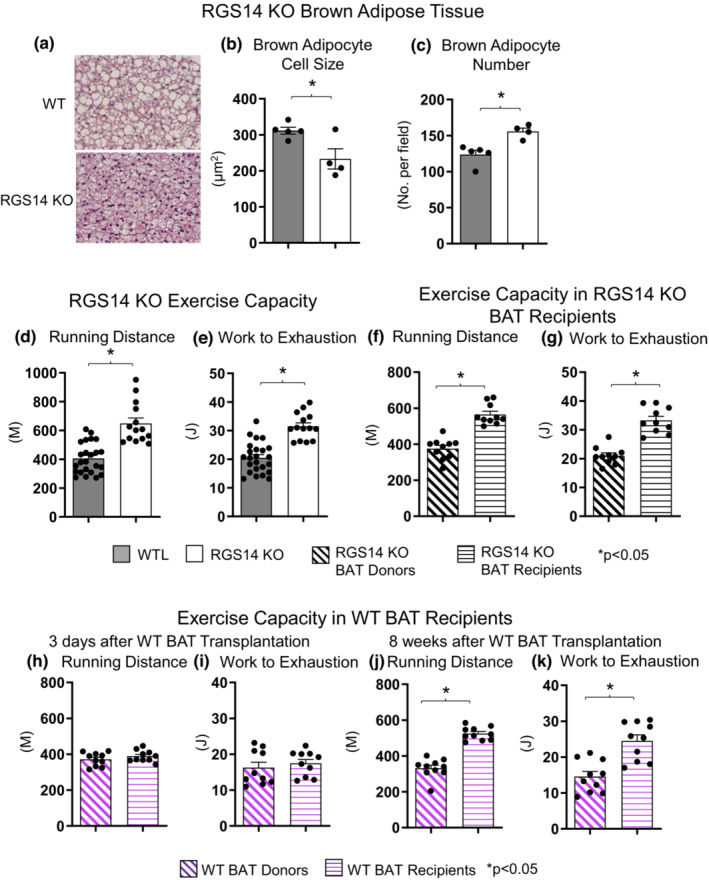
Increased Brown Adipose Tissue (BAT) cell numbers and increased exercise capacity in RGS14 KO mice. RGS14 KO mice exhibited smaller brown adipocytes (a, b), and increased number of brown adipocytes (a, c) than wild type (WT) control mice (*n* = 5 for WT, *n* = 4 for RGS14 KO). RGS14 KO mice ran longer distances (d) and did more work to exhaustion (e) compared to WT littermates (*n* = 24 for WT, *n* = 14 for RGS14 KO). BAT transplantation from RGS14 KO mice to WT mice led to a reversal of phenotype such that RGS14 KO BAT recipients exhibited improved running distance (f) and greater work to exhaustion (g) compared to RGS14 KO BAT donors, at 3 days after RGS14 KO BAT transplantation (*n* = 10 for each group). In contrast, there was no improvement in running distance and work to exhaustion at 3 days after transplantation of BAT from C57BL6/J WT mice to other C57BL6/J WT mice (h, i). *n* = 10 for each group. It required 8 weeks to achieve enhanced running distance and work to exhaustion in C57BI/6J WT mice with BAT transplantation from other C57BL6/J WT mice (j, k). *n* = 10 for each group. Results are expressed as Mean ± SEM. *, *p* < 0.05.

Our prior report on RGS14 KO mice revealed these mice to have improved body composition compared to WT littermates, that is, less white adipose tissue and body weight than WT (Vatner et al., 2018). Here, we report that RGS14 KO mice have smaller brown adipocytes (Figure 1a,b) along with greater numbers of brown adipocytes (Figure 1a,c).

### 3.1 RGS14 KO mice exhibit enhanced exercise capacity mediated by BAT

RGS14 KO mice ran significantly longer distances (648 ± 38 m vs. 405 ± 21 m; *p* < 0.05) and exhibited 53% increased work to exhaustion (*p* < 0.05) when compared to WT littermate controls (Figure 1d,e). After BAT was removed from RGS14 KO mice and transplanted to WT mice, the RGS14 KO BAT donors lost their enhanced running distance and work to exhaustion (Figure 1f,g), whereas these phenotypes were transferred to the WT mice with RGS14 KO BAT transplants. Within 3 days after BAT transplantation from RGS14 KO mice to WT mice, the RGS14 KO BAT recipients ran longer distances (565 ± 18 m vs. 375 ± 18 m; *p* < 0.05) (Figure 1f) and completed 58% more work to exhaustion (Figure 1g; *p* < 0.05) when compared to RGS14 KO BAT donors post‐BAT removal.

### 3.2 The salutary effect of transplanted RGS14 KO BAT arises more quickly than that of WT BAT

It normally requires 4–8 weeks for WT BAT to exert a salutary effect when transplanted to another WT mouse (Liu et al., 2013; Stanford et al., 2013). Indeed, in this study, enhanced exercise performance was not observed 3 days after transplantation of BAT from WT mice into WT mice (Figure 1h,i). Rather 8 weeks were required for BAT transplants from WT donor mice to enhance exercise capacity in WT recipient mice (Figure 1j,k).

### 3.3 RGS14 KO mice exhibit increased mitochondrial biogenesis mediated by SIRT3

**FIGURE 2:**
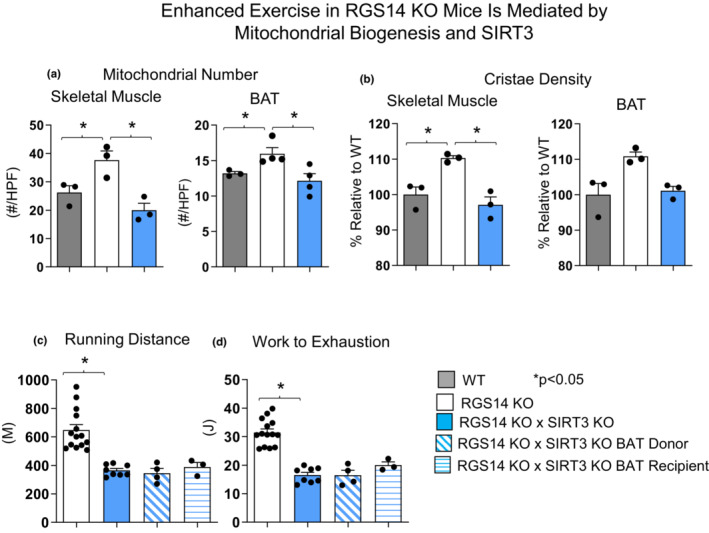
Enhanced exercise in RGS14 KO mice is mediated by mitochondrial biogenesis and SIRT3. RGS14 KO mice exhibited greater numbers of mitochondria (a) and higher cristae density (b) in both BAT and skeletal muscle, compared to WT mice, which were no longer observed when SIRT3 was disrupted in RGS14 KO mice (a, b). *n* = 3 for each group. The increased exercise capacity of RGS14 KO mice and mice with transplanted BAT from RGS14 KO was also no longer observed after disruption of SIRT3 in RGS14 KO mice (c, d). *n* = 14 for RGS14 KO mice, *n* = 8 for RGS14 KO × SIRT3 KO mice, *n* = 4 for RGS14 KO × SIRT3 KO BAT donors, *n* = 3 for RGS14 KO × SIRT3 KO BAT recipients. Results are expressed as Mean ± SEM. *, *p* < 0.05.

Analysis of the gastrocnemius muscles and BAT via transmission electron microscopy revealed higher mitochondrial number (Figure 2a) and cristae density (Figure 2b) in skeletal muscle and BAT tissue derived from RGS14 KO mice when compared to WT littermate controls, which was eliminated by SIRT3 disruption in RGS14 KO mice (Figure 2a,b). We previously reported increased expression of SIRT3 in RGS14 KO mice (Vatner et al., 2018). Exercise in RGS14 KO × SIRT3 KO mice revealed abolition of the enhanced exercise capacity seen in RGS14 KO mice, that is, running distance was reduced to 365 ± 14 m from 647 ± 38 m, *p* < 0.05, and work to exhaustion was reduced by 48%, *p* < 0.05, when compared to RGS14 KO mice (Figure 2c,d).

To further confirm the role of SIRT3 in modulating exercise capacity, BAT was transplanted from RGS14 KO × SIRT3 KO mice into WT mice. Transplantation from donor RGS14 KO × SIRT3 KO mice to WT recipient mice led to no improvement in running distance or work to exhaustion (Figure 2c,d).

### 3.4 Enhanced exercise capacity is mediated by improved metabolism in RGS14 KO mice

**FIGURE 3:**
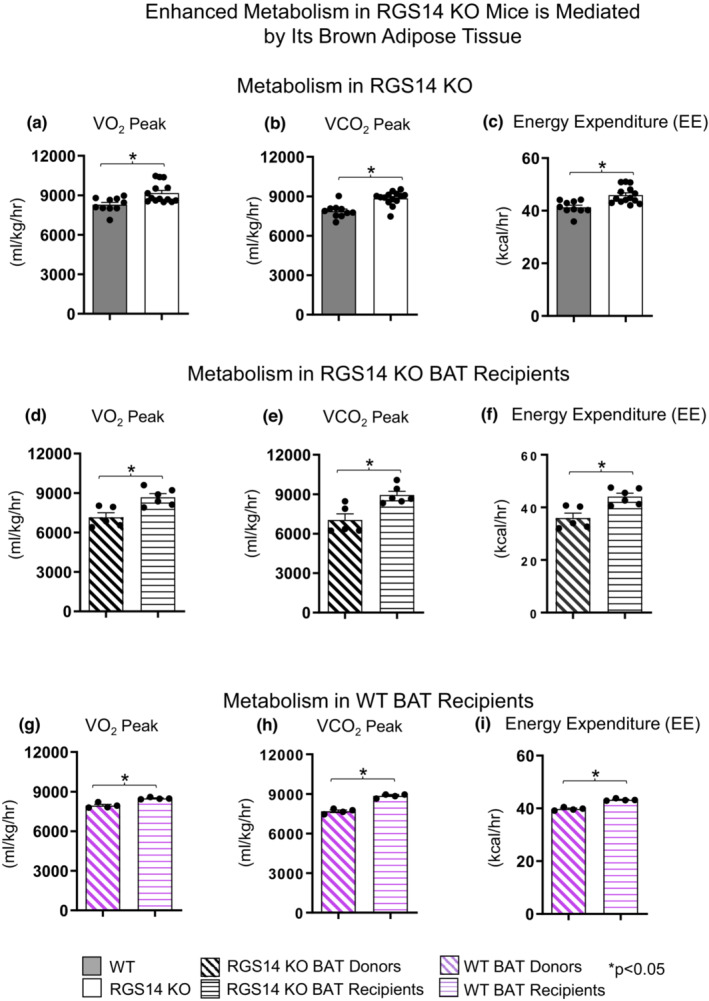
RGS14 KO mice and WT mice receiving RGS14 KO BAT exhibit enhanced metabolism. RGS14 KO mice compared to WT littermates exhibited higher maximal VO_2_ (a), VCO_2_ (b), and energy expenditure (c). BAT transplantation from RGS14 KO mice to WT mice led to a reversal of phenotype such that WT receiving BAT from RGS14 KO mice, at 3 days after transplantation, exhibited the same improved maximal VO_2_ (d), VCO_2_ (e), and energy expenditure (f) as was observed in RGS14 KO mice, but this improved metabolism was lost in the RGS14 KO BAT donors. The improved maximal VO_2_ (g), VCO_2_ (h), and energy expenditure (i) exhibited in WT BAT recipients was observed, not at 3 days after transplantation, but at 8 weeks after transplantation of BAT from C57BI/6J WT mice to other C57BL6/J WT mice. *n* = 10 for WT mice, *n* = 14 for RGS14 KO mice, *n* = 5 for RGS14 KO BAT donors, *n* = 6 for RGS14 KO BAT recipients. *n* = 4 for WT BAT donors, *n* = 4 for WT BAT recipients. Results are expressed as Mean ± SEM. *, *p* < 0.05.

Our previous RGS14 KO mouse study showed enhanced overall metabolism, as reflected by increased baseline VO_2_ in RGS14 KO mice mediated by BAT (Vatner et al., 2018). In the present investigation, indirect calorimetry was measured during exercise and VO_2_, VCO_2_, and EE were recorded at peak exercise capacity. RGS14 KO mice displayed higher VO_2_ peak (9175 ± 201 mL/kg/h vs. 8290 ± 172 mL/kg/h; *p* < 0.05), VCO_2_ peak (8849 ± 139 mL/kg/h vs. 7872 ± 165 mL/kg/h; *p* < 0.05), and EE (46 ± 0.9 mL/kg/h vs. 41 ± 1.0 kcal/kg/h; *p* < 0.05) when compared to WT littermate controls (Figure 3a–c). The data in RGS14 KO BAT recipients, 3 days after BAT transplantation, resembled that of RGS14 KO mice, and the data from RGS14 KO donor mice, resembled that of WT mice (Figure 3d–f). In addition, in this study, improved metabolism was observed 8 weeks after transplantation of BAT from WT mice into WT mice (Figure 3g–i).

### 3.5 Enhanced exercise capacity in RGS14 KO mice is also due to increased antioxidant defense

**FIGURE 4:**
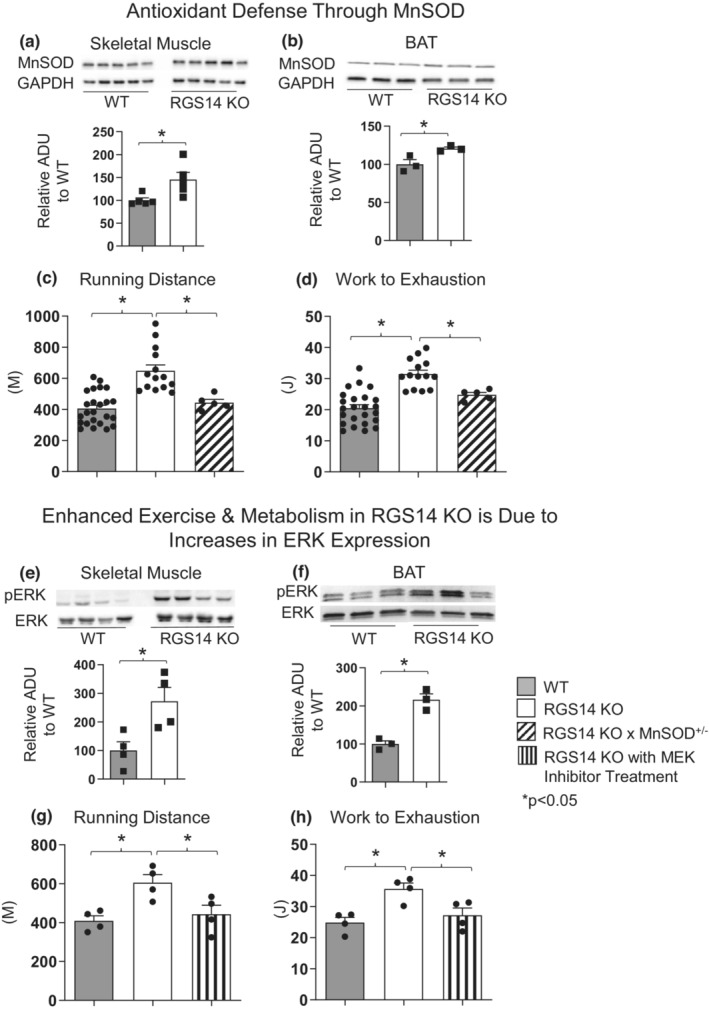
Enhanced exercise and metabolism in RGS14 KO mice is mediated by enhanced antioxidant defense and MEK/ERK signaling. RGS14 KO mice have higher expression of MnSOD in both skeletal muscle (a) and BAT (b) when compared to WT. *n* = 5 for each group of skeletal muscle, and *n* = 3 for each group of BAT. Genetic disruption of MnSOD expression in RGS14 KO mice led to decreases in running distance (c) and work to exhaustion (d). *n* = 24 for WT mice, *n* = 14 for RGS14 KO mice, *n* = 5 for RGS14 KO × MnSOD^+/−^ mice. pERK/ERK protein expression was increased in both the skeletal muscle (e) and BAT (f) of RGS14 KO mice. *n* = 4 for each group of skeletal muscle, *n* = 3 for each group of BAT. Enhanced running distance (g) and work to exhaustion (h) in RGS14 KO mice were both abolished after treatment with the MEK/ERK inhibitor U0126. *n* = 4 for each group. Results are expressed as Mean ± SEM. *, *p* < 0.05.

The protein level for manganese superoxide dismutase (MnSOD) was found significantly upregulated in both skeletal muscle and BAT of RGS14 KO mice (Figure 4a,b). We, therefore, examined the role of MnSOD in regulating the increased exercise capacity in RGS14 KO mice. To block the effects of MnSOD, we mated RGS14 KO mice with MnSOD heterozygous (MnSOD^+/−^) mice. Enhanced exercise capacity in RGS14 KO mice was abolished in RGS14 KO × MnSOD^+/−^ mice (Figure 4c,d).

### 3.6 Enhanced exercise capacity in RGS14 KO mice is mediated by the MEK/ERK pathway

RGS14 is not only a GTPase‐accelerating protein and guanine nucleotide dissociation inhibitor (GDI; (Kimple et al., 2002)) for heterotrimeric G‐alpha subunits (and, therefore, a negative regulator of G protein‐coupled receptor [GPCR] signaling), but this protein is also a mitogen‐activated protein kinase (MAPK) scaffold that coordinates multiple components of the MEK/ERK signal transduction cascade (Vellano et al., 2013). This latter regulatory function of RGS14 is thought to be important for its observed effect on mouse CNS signaling in learning and memory circuitry; mice lacking RGS14 expression exhibit enhanced learning and memory, and this enhancement can be eliminated by administration of a MEK inhibitor (Lee et al., 2010). However, RGS14 has not previously been shown to modulate exercise performance through the MEK/ERK signaling pathway. We examined the MEK downstream molecule, ERK, by western blotting and found that phosphorylation of ERK was significantly increased in the skeletal muscle and BAT of RGS14 KO (Figure 4e,f). Our data demonstrate that the MEK/ERK pathway is also involved in the enhanced exercise capacity of RGS14 KO mice, as this enhancement of exercise capacity was abolished by treatment with a MEK inhibitor, U0126 (Figure 4g,h).

### 3.7 Enhanced exercise capacity is mediated by BAT through angiogenesis and arteriogenesis resulting in increased Hindlimb blood flow

**FIGURE 5:**
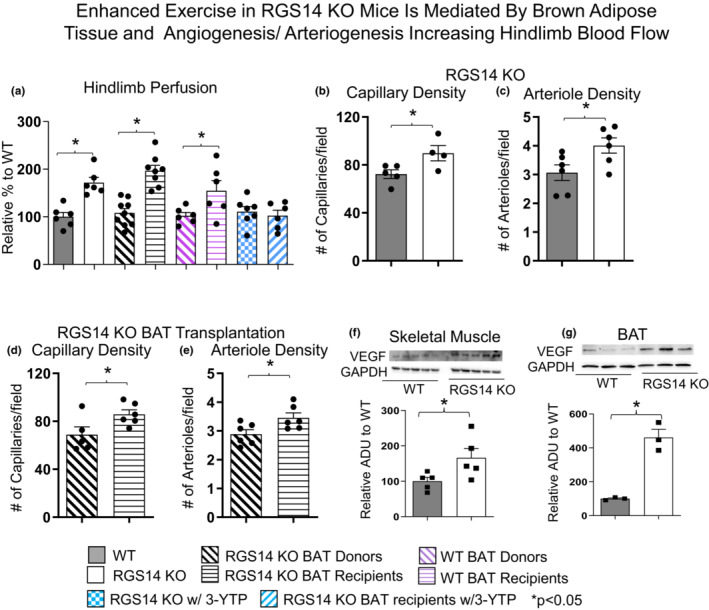
Enhanced exercise by RGS14 KO mice is mediated by BAT and angiogenesis/arteriogenesis increasing Hindlimb blood flow. Non‐linear contrast imaging was used to measure hindlimb blood flow. The average data are presented as % of WT perfusion, which is represented as 100% (a). Hindlimb blood flow was higher in RGS14 KO mice compared to WT mice, and higher in WT mice that received RGS14 KO BAT, at 3 days after transplantation (a, b), while RGS14 KO BAT donors lost their enhanced hindlimb perfusion, with results similar to WT mice (a). With treatment of the SIRT3 inhibitor, 3‐YTP, both RGS14 KO mice and RGS14 KO BAT recipients lost their enhanced hindlimb perfusion (a). WT BAT recipients showed greater hindlimb perfusion at 8 weeks after transplantation of BAT from C57BL6/J WT mice to other C57BL6/J WT mice (a). *n* = 6 for WT mice, *n* = 6 for RGS14 KO mice, *n* = 9 for RGS14 KO BAT donors, *n* = 8 for RGS14 KO BAT recipients, *n* = 6 for WT BAT donors, *n* = 6 for WT BAT recipients, *n* = 7 for RGS14 KO mice + 3‐YTP, *n* = 6 for RGS14 KO BAT recipients + 3‐YTP. Angiogenesis (reflected by capillary density) and arteriogenesis (reflected by arteriole density) were both increased in skeletal muscle of RGS14 KO mice (b, c) and RGS14 KO BAT recipients (d, e), which correlated with increased VEGF in skeletal muscle (f) and BAT (g). Increased angiogenesis (d) and arteriogenesis (e) were not observed in RGS14 KO BAT donors. For capillary density: *n* = 5 for WT mice, *n* = 4 for RGS14 KO mice, *n* = 5 for RGS14 KO BAT donors, *n* = 6 for RGS14 KO BAT recipients. For arteriole density: *n* = 6 for each group. For VEGF: *n* = 5 for each group of skeletal muscle, *n* = 3 for each group of BAT. Results are expressed as Mean ± SEM, *, *p* < 0.05.

Skeletal muscle blood flow was measured in the hindlimb in both RGS14 KO and WT mice. RGS14 KO mice exhibited 72 ± 11% increased perfusion in the hindlimb compared to WT (Figure 5a, left panel). After BAT removal, RGS14 KO BAT donor mice experienced reduced perfusion, similar to that observed in WT, while WT recipients of RGS14 KO BAT exhibited increased perfusion, at 3 days after BAT transplantation, similar to that observed in RGS14 KO (Figure 5a). However, it took 8 weeks after BAT transplantation from WT mice, for WT BAT recipients to exhibit greater hindlimb perfusion than WT BAT donors (Figure 5a). In addition, the enhanced hindlimb blood flow seen in RGS14 KO mice, and RGS14 KO BAT recipients was abolished by a SIRT3 inhibitor, 3‐YTP (Shi et al., 2022; Ye et al., 2019) treatment (Figure 5a).

In addition, greater angiogenesis was found in RGS14 KO mice and in RGS14 KO BAT recipients' skeletal muscle, as reflected in greater capillary density (Figure 5b,d). Similar results for arteriogenesis and arteriole density were found (Figure 5c,e). Increased levels of the vascular growth factor VEGF were found in RGS14 KO mice in both skeletal muscle and BAT (Figure 5f,g).

## 4 DISCUSSION

Discovery of novel mechanisms promoting improved exercise performance is important given that exercise can help prevent and alleviate several different diseases, most notably, cardiovascular disease, cancer, and obesity and is a key factor in healthful longevity (Brown et al., 2012; Craft & Perna, 2004; McQueen, 2009; Myers, 2003). Here, we have presented that the genetic disruption of RGS14 is a novel model of enhanced exercise capacity, such that RGS14 KO mice run longer distances and do more work than WT littermate controls. Furthermore, RGS14 KO mice exhibit improved metabolism through increases in peak VO_2_, VCO_2_, and EE, and enhanced hindlimb blood flow accompanied with increases in VEGF. Also, the RGS14 KO mice have enhanced BAT function, which mediates the enhanced exercise capacity. Furthermore, the improved metabolism observed in RGS14 KO mice, as measured by VO_2_ and VCO_2_ peaks, must be due to enhanced BAT function, as activated BAT has previously been shown to lead to increases in body temperature and metabolic rate (Coombes et al., 1987).

BAT transplantation from RGS14 KO mice into WT mice further confirmed the role of BAT mediating the enhanced exercise capacity in RGS14 KO mice, given that RGS14 KO BAT donors lose their enhanced exercise benefits post‐transplantation (soon after BAT loss), while WT mice receiving RGS14 KO BAT recapitulate the enhanced exercise performance observed in RGS14 KO mice, at 3 days after BAT transplantation. Similar to intact RGS14 KO mice, the RGS14 KO BAT recipients exhibit similar BAT morphology after transplantation, suggesting that the BAT itself is intrinsically altered by the absence of RGS14 and mediates changes to exercise capacity directly. BAT transplanted from WT to WT mice also improved exercise performance, but only at 8 weeks after transplantation, as opposed to 3 days after BAT transplantation from RGS14 KO mice.

Whereas the current investigation focused on mechanisms mediating enhanced exercise performance by BAT and by disruption of RGS14, there are several studies demonstrating that exercise can affect BAT regulation. It is known that exercise and chronic exercise training can change BAT tissue function (Agarwal, 2012; Aldiss et al., 2018; Cuevas‐Ramos et al., 2019; Dewal & Stanford, 2019), most notably conversion of white adipose tissue to a functionally equivalent BAT subtype (Aldiss et al., 2018; Dewal & Stanford, 2019) composed of the “beige” adipocyte with an intermediate phenotype, but results regarding the effects of these changes are conflicting. Some studies have reported exercise‐induced improvement in BAT mitochondrial function (Ignacio et al., 2012; Yoshioka et al., 1989), while others have found the opposite result (Sanchez‐Delgado et al., 2016; Vosselman et al., 2015; Wu et al., 2014).

While prior studies have examined the effects of exercise on BAT function, the reverse scenario (i.e., potential effects of BAT on exercise capacity) has been essentially ignored, despite the fact that transplantation of normal BAT is known to confer multiple beneficial effects, most notably anti‐obesity and anti‐diabetes actions, such as improved glucose metabolism and insulin sensitivity (White et al., 2019). One study previously assessed whether BAT influences exercise capacity (Kong et al., 2018), but, in contrast to our data, this prior study suggested that BAT might contribute negatively to skeletal muscle performance (Kong et al., 2018). In that study, BAT from interferon regulator factor‐4 (IRF4)‐deficient mice was reported to produce and secrete the potent factor myostatin, which is well documented as a negative regulator of skeletal muscle cell differentiation (Kong et al., 2018). Conversely, our results suggest that the RGS14 KO model ‐ a model with enhanced BAT ‐ exhibits greater exercise capacity and improved metabolism.

One of the most important regulators of BAT function is SIRT3, a mitochondrial sirtuin deacetylase. SIRT3 regulates the expression of many BAT mitochondrial proteins including UCP‐1 (Sebaa et al., 2019) and, therefore, may play a large role in mediating the enhanced BAT and, consequently, the enhanced exercise capacity of RGS14 KO mice. SIRT3 is highly expressed in “master athletes” as well as those that engage in exercise training (Koltai et al., 2018). SIRT3 is also upregulated with exercise in animal models (Cheng et al., 2016). We have previously shown that the protein levels of SIRT3, not SIRT1, are upregulated in the BAT and skeletal muscle of RGS14 KO mice (Vatner et al., 2018), and therefore, SIRT3 is considered an important regulator of exercise capacity in this model. Since SIRT3 is a mitochondrial protein, we decided to analyze the ultrastructure of mitochondria in RGS14 KO and WT mice. RGS14 KO mice exhibited increases in mitochondrial number and cristae density, supporting a role for SIRT3 in mediating mitochondrial biogenesis. The analysis of mitochondria in RGS14 KO × SIRT3 KO mice showed a reversion to similar parameters observed in WT mice. These changes correlated with exercise capacity, such that RGS14 KO × SIRT3 KO mice did not show the enhanced exercise capacity of RGS14 KO mice. The role of SIRT3 in BAT function was confirmed by demonstrating that the enhancement of exercise capacity upon RGS14 KO BAT transplantation to WT mice was not observed when BAT was transplanted from RGS14 KO × SIRT3 KO mice, even 6 months after transplantation. This confirms that SIRT3 is necessary for the enhanced exercise capacity seen in the RGS14 KO mouse and conferred by BAT transplantation.

Skeletal muscle is an “on‐demand”, highly metabolic tissue, and thus a natural byproduct of exercise is reactive oxygen species. Better reactive oxygen species management enhances exercise endurance, which underscores the reciprocal relationship between oxidative stress and skeletal muscle metabolism (Simioni et al., 2018). SIRT3 is known to mediate protection from oxidative stress and mitochondrial function and enhance exercise capacity (Lin et al., 2014). MnSOD is a primary mitochondrial ROS scavenging enzyme, which can be activated by SIRT3 (Qiu et al., 2010). SIRT3 has already been shown to regulate MnSOD directly such that knockdown of SIRT3 leads to an increase in superoxide levels through decreased MnSOD activation (Chen et al., 2011). There are several studies showing the relationship between SIRT3 and MnSOD (SOD2) and improved exercise; showing both that SIRT3 can improve exercise performance and conversely that exercise can lead to increased SIRT3 (Cheng et al., 2016; Cho et al., 2022; Zhou et al., 2022). Therefore, the RGS14 KO mouse, which exhibits increased MnSOD activity, which also contributes to its enhanced exercise capacity, further confirmed with partial genetic ablation of MnSOD, which abolished the enhanced exercise capacity. Furthermore, this outcome is consistent with another study, showing disruption of RGS14 in rat fibroblasts leads to increased resistance to oxidative stress involving higher MnSOD expression (Lin et al., 2011).

RGS14 itself is also known to directly affect MAPK/ERK signaling, and therefore, its absence could also lead to elevation in intrinsic MAPK/ERK signal transduction following upstream activation events such as Ras‐family GTPase activation (Vellano et al., 2013). Additionally, the MEK/ERK pathway is involved in angiogenesis/arteriogenesis (Chim et al., 2014; Deng et al., 2013). We found that the MEK/ERK pathway is also involved in the enhanced exercise capacity of RGS14 KO mice, as this enhancement of exercise capacity was abolished by treatment with a MEK inhibitor, U0126 (Figure 4e,f). Although a recent article on RGS14 suggested that overexpression of RGS14 was beneficial to the heart through the MEK/ERK signaling pathway (Li et al., 2016), there are no prior studies linking MEK/ERK and SIRT3 on exercise capacity, except a study showing both MEK/ERK and SIRT3 are involved in amyloid beta proteins in brain (Cieslik et al., 2015). In our previous study on another healthful longevity model, adenylyl cyclase type 5 (AC5) KO mice, we showed that MnSOD is a downstream target of MEK/ERK pathway (Lai et al., 2013), and mediates enhanced exercise capacity in AC5 KO mice (Vatner et al., 2015).

Another powerful mediator of exercise performance is blood flow. We found here that RGS14 KO mice exhibit enhanced hindlimb blood flow (Figure 5a), which is accompanied by increases in angiogenesis and arteriogenesis in the hindlimb vasculature leading to increased capillary and arteriole density (Figure 5a–e). This increased angiogenesis, arteriogenesis and blood flow are likely due to angiogenic factors, the most potent being VEGF. A direct connection between VEGF and BAT has already been established, as VEGF is known to play a direct and positive role in the activation and expansion of BAT (Sun et al., 2014). VEGF also acts in an endocrine and paracrine manner in BAT by stimulating the proliferation of vascular endothelial cells (Bagchi et al., 2013). RGS14 KO mice have increased VEGF expression in the skeletal muscle and BAT (Figure 5f,g). Removing BAT from RGS14 KO mice resulted in loss of the significant increase in hindlimb perfusion, while the addition of RGS14 KO BAT to WT mice led to increases in perfusion. This addition also led to changes in the vasculature, with RGS14 KO BAT recipients exhibiting increases in capillary and arteriole density.

**FIGURE 6:**
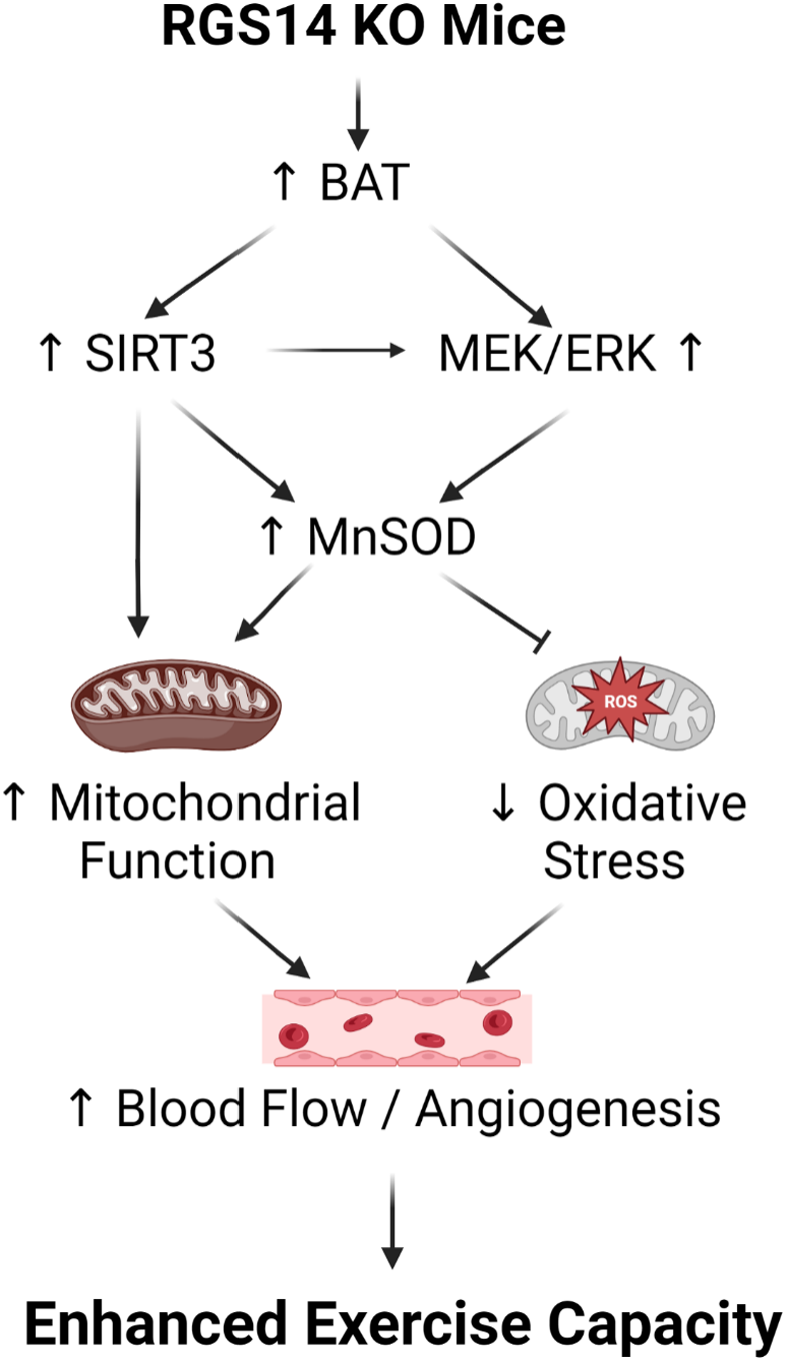
Mechanisms mediating enhanced exercise capacity in RGS14 KO and its uniquely powerful BAT. Multiple mechanisms mediated the enhanced exercise capacity in RGS14 KO mice. The most important mechanism is brown adipose tissue (BAT), which mediates SIRT3, MnSOD, MEK/ERK and VEGF pathways. These mechanisms regulate exercise capacity by improved mitochondrial function, protection against oxidative stress and improved blood flow/angiogenesis.

In summary, we found several mechanisms mediating the enhanced exercise performance in RGS14 KO mice and WT mice with RGS14 BAT transplants, that is, SIRT3, MEK/ERK, MnSOD, and decreased oxidative stress resulting in increased skeletal muscle blood flow and angiogenesis (Figure 6). Although our findings that BAT mediates enhanced exercise performance through these mechanisms is novel, a study has shown that transplanted BAT from C57B/L6 mice becomes revascularized (Stanford et al., 2013), and receiving BAT from Fat‐1 transgenic mice upregulates VEGF levels in endogenous BAT (Tsuji et al., 2022). However, neither of these studies examined blood flow regulation. Moreover, a recent study found the opposite, that is, that the transplanted BAT derived from C57B/L6 mice did not improve blood flow or VEGF levels in HFD‐fed mice (Chen et al., 2018). Another study found increased phosphorylated ERK levels in Ob/Ob mice after receiving BAT from C57B/L6 mice (Liu et al., 2015), but did not link it to effects of exercise. There are also no prior studies showing that BAT regulates SIRT3 or MnSOD or oxidative stress.

In conclusion, the major findings of this investigation are that BAT from WT mice improves exercise performance, and that BAT from RGS14KO mice is unique and a more powerful regulator of exercise performance than BAT from WT mice. Multiple mechanisms mediated the enhanced exercise due to BAT, most notably the SIRT3 pathway and improved metabolism and mitochondrial biogenesis. In addition, angiogenesis and arteriogenesis, resulting in increased capillary and arteriole density, mediating increased blood flow during exercise also contributed, as well as increased antioxidative defense and MEK/ERK signaling. The results of the current investigation provide novel information of BAT's regulation of exercise performance and should provide new pathways and therapeutic modalities to promote healthful aging.

## AUTHOR CONTRIBUTIONS

S.F.V and D.E.V. designed the study. S.F.V., D.E.V., and M.O. conducted the study. S.F.V., D.E.V., M.O., J.Z., and S.C.C. wrote the manuscript.

## ACKNOWLEDGEMENTS

We appreciate Dr. Chujun Yuan for technical support.

## FUNDING INFORMATION

This study was supported by National Institutes of Health grants R01HL106511, R01HL124282, R01HL137368 (to S.F.V.), S10OD025238 (to S.F.V. for Vevo 3100), and R01HL137405 (to D.E.V.).

## CONFLICT OF INTEREST STATEMENT

None declared.

## Data Availability

The data generated or analyzed during this study and included in this published article will be available upon reasonable request to the corresponding author.
